# Progress in the Development of Active-Matrix Quantum-Dot Light-Emitting Diodes Driven by Non-Si Thin-Film Transistors

**DOI:** 10.3390/ma15238511

**Published:** 2022-11-29

**Authors:** Geun Woo Baek, Yeon Jun Kim, Minhyung Lee, Yeunwoo Kwon, Beomsoo Chun, Ganghyun Park, Hansol Seo, Heesun Yang, Jeonghun Kwak

**Affiliations:** 1 Department of Electrical and Computer Engineering, Inter-University Semiconductor Research Center, Soft Foundry Institute, Seoul National University, Seoul 08826, Republic of Korea; 2 Department of Materials Science and Engineering, Hongik University, Seoul 04066, Republic of Korea

**Keywords:** active-matrix, quantum-dot light-emitting diodes, thin film transistors, metal oxides, transition metal dichalcogenides, carbon nanotubes

## Abstract

This paper aims to discuss the key accomplishments and further prospects of active-matrix (AM) quantum-dot (QD) light-emitting diodes (QLEDs) display. We present an overview and state-of-the-art of QLEDs as a frontplane and non-Si-based thin-film transistors (TFTs) as a backplane to meet the requirements for the next-generation displays, such as flexibility, transparency, low power consumption, fast response, high efficiency, and operational reliability. After a brief introduction, we first review the research on non-Si-based TFTs using metal oxides, transition metal dichalcogenides, and semiconducting carbon nanotubes as the driving unit of display devices. Next, QLED technologies are analyzed in terms of the device structure, device engineering, and QD patterning technique to realize high-performance, full-color AM-QLEDs. Lastly, recent research on the monolithic integration of TFT–QLED is examined, which proposes a new perspective on the integrated device. We anticipate that this review will help the readership understand the fundamentals, current state, and issues on TFTs and QLEDs for future AM-QLED displays.

## 1. Introduction

Colloidal quantum dots (QDs) have been regarded as promising emitters owing to their excellent optical properties, such as near-unity photoluminescence (PL) quantum yield (QY), narrow emission spectral bandwidth, and size-dependent bandgap tunability. Based on these merits, QDs can be utilized for a variety of applications, such as display devices, solar cells, and photodetectors [1,2,3,4,5,6,7,8,9,10]. In particular, QDs have been widely investigated for their use in display devices to improve the color gamut. For instance, a liquid crystal display (LCD), including a QD-based color conversion film, has already been commercialized. Meanwhile, interest in QD-based light-emitting diodes (QLEDs) has been increasing for decades to take advantage of their superb optoelectronic performance, solution-processability, and fabrication compatibility with existing technologies, which enabled QLEDs to exhibit high efficiency, luminance, and stability comparable with those of organic light-emitting diodes (OLEDs) [11,12,13,14,15,16]. Additionally, the demonstration of active-matrix (AM) QLED displays using a Si-based thin-film transistors (TFTs) backplane has been raising the expectation for high-quality displays [17,18,19,20,21,22]. Despite the remarkable development, however, further research on AM-QLEDs driven by non-Si-based TFTs is required to realize display devices with advanced features, e.g., transparency, flexibility, and stretchability, which has rarely been reported so far [23,24,25,26]. This may be because the TFT backplane needs to meet more stringent requirements, such as good electrical properties to drive high current, low response to light from QLED, and endurance to the solution-based QLED fabrication processes [27,28,29,30,31].

In this review, we aim to provide recent progress of non-Si-based TFTs and QLEDs for AM-QLED displays. In the first section, we briefly introduce the importance of using non-Si-based TFT backplane, the materials and technical issues of these TFTs using metal oxides, transition metal dichalcogenides (TMDCs), and semiconducting single-walled carbon nanotubes (SWNTs). Next, recent research on QLEDs as a frontplane device is reviewed in terms of device engineering, including the device architecture for QLEDs, device modification for performance improvements, and fine-patterning methods for QD films. Lastly, prospects and remaining challenges in research of TFT-driven AM-QLED displays will be discussed.

## 2. Non-Si TFT Backplane Technology

**Figure 1:**
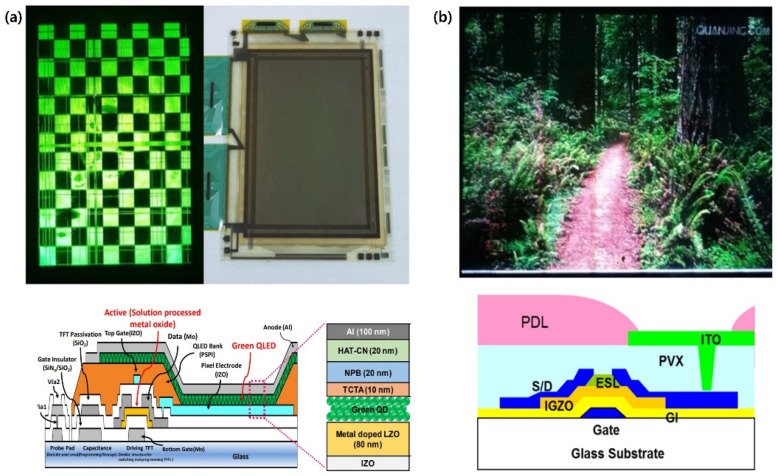
AM-QLEDs driven by various oxide TFTs. (**a**) Green-emitting 4-inch display driven by solution-processed Li-doped ZnO TFTs and its single-pixel structure (reproduced from [32] with permission from Wiley). (**b**) Full-color 14-inch display driven by IGZO TFTs and its single-pixel structure (reproduced from [22] with permission from Wiley).

An AM backplane consists of at least two types of TFTs: a driving TFT to supply current to a subpixel and a switching TFT to turn on and off the addressed driving TFT. Therefore, the performance of backplane TFTs is important to properly provide signals and currents to subpixels. It is typically required for TFT devices to have good mobility for fast response, large on-current for high brightness, low leakage current for low power consumption, and high reliability for stable operation. For the AM-LCDs, amorphous Si is mostly used because it does not require high mobility to switch the subpixels. In the case of AM-OLED-based products, including mobile phones and televisions, low-temperature poly-Si (LTPS) is used to quickly drive a subpixel with sufficient current. However, these Si-based TFTs cannot be used for flexible and transparent displays. Thus, presently, n-type oxide TFTs, for example, indium gallium zinc oxide (IGZO), exhibiting high electron mobility (*µ*_e_) of >10 cm^2^ V^−1^ s^−1^ and decent operational stability, are adopted for large-area and foldable AM-OLED displays. Various n-type oxide TFTs have also been utilized for AM-QLEDs [20,21,24,32], as shown in Figure 1. Nevertheless, p-type TFTs are also needed to comply with various QLED architectures (e.g., normal and inverted structures) and solution-based QLED fabrication processes. In this regard, TMDCs and semiconducting SWNTs exhibiting high hole mobility (*µ*_h_) can be good candidates for p-type channel materials. In this section, we explain a fundamental circuit design for the AM driving mode, state-of-the-art, and remaining challenges of non-Si-based TFT backplane technologies for AM-QLEDs.

### 2.1. Circuits for AM Driving

**Figure 2:**
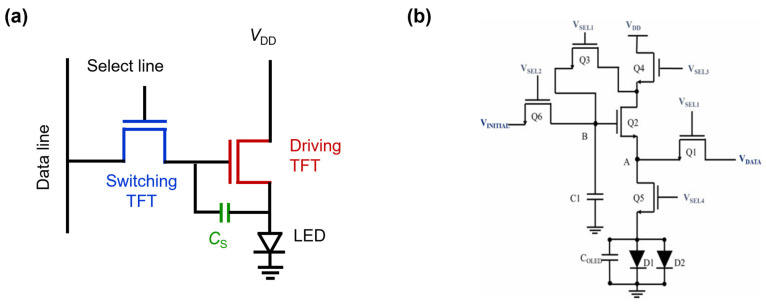
Schematic illustration for AM driving: (**a**) Typical 2T1C circuit and (**b**) 6T1C pixel compensation circuit (reproduced from [33] with permission from Elsevier).

As is well-known, a passive-matrix-driven display requires a regular scanning signal with a high frequency to make a subpixel be perceived as a turn-on state, which consumes a large amount of power. On the contrary, with the AM driving circuit that basically consists of two TFTs and one storage capacitor (*C*_S_) for a single pixel (i.e., the 2T1C structure), the light-emitting diode (LED) can continuously emit light even when the switching TFT has no signal, as shown in Figure 2a because *C*_S_ can store the on/off state by holding the gate voltage of the driving TFT. Thus, it is essential to use the AM driving circuit for low power consumption and faster switching in the latest display devices.

In spite of these advantages, the AM driving method has a critical issue of non-uniform degradation of LEDs and TFTs in each pixel. In detail, LEDs may have different lifetimes for each individual color of subpixels. Similarly, TFTs suffer from uneven threshold voltage (*V*_T_) shifts originating from bias–temperature stress (BTS). In order to alleviate these phenomena, various compensation circuits have been proposed, including voltage (or current) programmable circuits and hybrid-type programmed circuits [33,34,35,36]. However, compensation circuits generally require more TFTs and capacitors, as exemplified in Figure 2b, which makes the TFT design and process steps complicated and increases the manufacturing cost of TFT backplanes. Therefore, TFTs with long-term reliability, high uniformity, and high performance needs to be developed continuously.

### 2.2. Active Materials for Non-Si TFT Backplane

In order to satisfy the aforementioned conditions, promising candidates have been researched, including metal oxides, TMDCs, and semiconducting SWNTs. Among these, oxide TFTs exhibit higher mobility than amorphous Si TFTs and improved uniformity in large-sized arrays than LTPS TFTs. Owing to these merits, oxide TFT-based AM-OLED displays have been successfully commercialized. However, the electron mobility of oxide TFTs needs to be improved further for a high scanning rate and high brightness by driving QLEDs with high currents. So far, researchers have reported several methods to increase the electrical properties of the oxide-based channel via tuning the chemical composition [37], post-treatment [38], a multilayered channel [39], a dual gate structure [40], and a high-*k* dielectric [41,42]. Based upon these efforts, Magari et al. recently reported a hydrogenated polycrystalline In_2_O_3_ TFT exhibiting a high *µ*_e_ of 139.2 cm^2^ V^−1^ s^−1^ by controlling the grain size and reducing defect sites [43].

However, oxide TFTs hardly operate in p-type. The integration of an n-type TFT and a normal structured QLED can cause an intricate fabrication procedure for wiring the drain electrode of the TFT with the top cathode of the QLED for electron injection. It may also increase the resistance causing Joule heating, which thus can reduce the response speed and degrade the reliability of the TFT backplane. Therefore, the development of p-type TFTs is necessary for high-performance AM-QLEDs. Previously, oxides were also one of the p-type channel candidates. However, p-type oxides, such as NiO, SnO, and CuO, mostly suffer from the lack of stability and possess an anisotropic, localized O 2p orbital with a large hole effective mass, leading to *µ*_h_ of <1 cm^2^ V^−1^ s^−1^ [44,45,46,47].

As an alternative, TMDCs in a chemical composition of MX_2_, where M is a transition metal (Mo, W, etc.) and X is a chalcogen (S, Se, or Te), have been focused as a promising p-channel material owing to their advanced properties, such as tunable bandgap depending on the number of layers, absence of dangling bonds, and resultingly a high on–off ratio with decent mobility. In addition, it is possible to form a functional heterostructure of two different materials. Among various TMDCs, MoTe_2_ can be a good choice for a TFT backplane because of its a few distinctive characteristics, i.e., insensitivity to light, easy type-convertibility, and bias stress instability [23,25,48]. On the other hand, exfoliation-based deposition and fine-patterning of the TMDC monolayer are the major obstacles to their application in large-area AM displays. Although technologies for high-resolution and large-scale fabrication have consistently progressed [49,50,51], they need to be developed more to satisfy customer expectations for large-sized displays.

For the large-area processability, semiconducting SWNTs would be an option for the TFT channel. The SWNT-based TFTs are easily fabricated using various solution processes at low temperatures, and they also show high device performance with intrinsic field-effect mobility (*µ*_h_ of up to ~10^2^ cm^2^ V^−1^ s^−1^), flexibility, and transparency [52,53,54,55]. They are also resistant to the subsequent processes for patterning and QLED deposition. Contrary to the advantages, there are a few known issues of SWNTs, such as low chirality and poor dispersity in solution—though the chirality is improving gradually, reaching 99.999% or even higher purity through post-treatment [56,57,58], and the dispersity has been enhanced by wrapping with DNA or conjugated polymers [59,60,61,62]. Poor n-type performance of SWNTs also needs to be enhanced for practical use in a display backplane, to be combined with an inverted QLED. A few of the TFT performances using oxides, TMDCs, and SWNTs reported within five years are summarized in Table 1.

Organic semiconductors have also been considered as active channel materials. Several small molecules (e.g., pentacene, thienothiophene derivatives) and polymers have been studied owing to their low cost and facile fabrication with high scalability [63,64,65]. However, it has limitations to employ as backplane TFTs due to its low mobility, high threshold voltage, low BTS, and patterning issues [66,67]. These problems need to be overcome for their practical use in backplanes.

**Table 1:** Summary of TFT parameters using oxides, TMDCs, and SWNTs.

Year	Channel Material	Type	Mobility (cm^2^ V^−1^ s^−1^)	Subthreshold Swing (V dec^−1^)	I_on/off_ Ratio	Threshold Voltage (V)	Reference
2018	IGZO	n	61.3	0.08	~10^6^	0.78	[68]
	MoS_2_	n	260	-	~10^6^	-	[69]
	SWNT	n	10	-	~10^8^	-	[70]
2019	IGZO	n	74	0.21	~10^8^	−1.3	[41]
	MoS_2_	n	170	-	~10^6^	-	[71]
	SWNT	p	20.9	0.2	~10^4^	0.4	[72]
2020	ZnON	n	147	0.21	~10^4^	−0.72	[40]
	MoS_2_	n	107	-	~10^6^	-	[73]
	SWNT	p	16	-	-	-	[74]
	MoTe_2_	p	178.7	-	~10^7^	−22	[75]
2021	IGTO *	n	116.5	0.13	~10^9^	0.47	[76]
	MoS_2_	n	44.6	0.36	~10^5^	-	[77]
	SWNT	p	10.92	0.4	~10^5^	0.48	[78]
	MoTe_2_	p	12.6	0.26	~10^4^	0.87	[79]
2022	In_2_O_3_:H	n	139.2	0.19	-	0.2	[43]
	MoS_2_	n	12.3	-	~10^9^	-	[80]
	SWNT	p	496	-	~10^8^	-	[81]
	MoTe_2_	p	30	-	~10^6^	−20	[82]

* Indium-Gallium-Tin-Oxide.

## 3. QLED Frontplane Technology

A QLED is a device for utilizing electroluminescence (EL) from QDs, consisting of hole injection/transport layers (HIL/HTL), electron injection/transport layers (EIL/ETL), and a QD emissive layer between them, similar to an OLED structure. Since the first QLED was reported [83], the efficiency and operational lifetime have been improved owing to the advances in QD synthesis, surface treatment, device architecture, and fundamental understandings of the QLEDs [84]. To realize the AM-QLED display, QLEDs should have high efficiency and brightness for low power consumption and a long operational lifetime for maintaining image quality. High-resolution patterning of the QD layer is also required for future display devices. Improvement of electron–hole balance in a QLED is the most significant issue to be solved because it is directly related to several kinds of non-radiative recombination processes (e.g., Auger recombination and exciton–polaron quenching) which affect the QLED efficiency and efficiency roll-off. Despite multilateral efforts, it is not easy to fundamentally overcome this issue due to a few inherent characteristics of QDs and QLEDs—an extremely narrow exciton recombination zone [85] and large energy level offsets with poor interfacial morphology at the QD–organic interface. According to the device structure and process compatibility, the integration of a QLED with a driving TFT needs to be studied. In this section, we briefly review the progress of QLEDs toward high-performance AM-QLEDs from the viewpoints of the device structure and engineering of the QDs and charge transport layers for improved electron–hole balance.

### 3.1. Device Architecture for QLEDs

**Figure 3:**
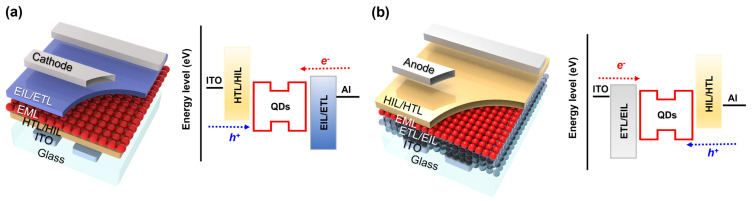
QLED device architecture with a schematic energy band diagram: (**a**) A normal structure and (**b**) an inverted structure.

The device architecture of QLEDs can be classified in a number of ways. According to the stacking order of each functional layer, QLEDs can be distinguished into two types, a normal structure (also called a conventional or standard structure) and an inverted structure. Figure 3a shows a normal QLED structure with a schematic energy band diagram, which is composed in a sequence of the anode, HIL, HTL, QDs, ETL, EIL, and cathode from the substrate. Because solution-based QD deposition is likely to damage the underlying organic HIL/HTL layers, the selectivity of charge-transporting materials and fabrication steps are significantly influenced in this structure. Also, an HTL possessing a deep, highest-occupied-molecular-orbital (HOMO) energy level is favorable for hole injection from organic HTL to inorganic QDs due to a large energy barrier between them. On the contrary, an inverted QLED is produced in the opposite order, from the cathode at the bottom to the anode on the top, as depicted in Figure 3b. As reported previously [86], this structure is beneficial for electron injection from the bottom cathode to QDs by adopting metal oxide nanoparticles (e.g., ZnO, ZnMgO, SnO, etc.) as the ETL with resistance to the follow-up QD deposition process. They have a good *µ*_e_ of approximately 10^−4^–10^−3^ cm^2^ V^−1^ s^−1^ and well-alignment of the conduction band level with QDs [86]. The relatively high charge density of metal oxides can transfer electrons to negatively charge QDs even without bias, facilitating electron injection into QDs by Auger-assisted carrier injection [87,88]. It also allows the expanded choice of HTL materials with the desired energy levels and mobility for boosting the hole injection using various deposition methods. Lastly, the stacking order has an influence on QLED–TFT integration. Considering that QLED fabrication starts on a substrate with a TFT array, it is more propitious to combine a normal QLED with a p-type TFT to supply hole current to the bottom anode or, similarly, an inverted QLED with an n-type TFT.

Depending on the direction of light emission, QLEDs are divided into bottom- and top-emission structures, which should be taken into consideration when designing the AM-QLEDs. In the case of the bottom-emission structure, the AM-QLED backplane must be transparent because the light emitted from the QLED subpixel needs to travel through the substrate. But as opaque metal wiring and TFTs below QLEDs reduce the aperture ratio, the bottom-emission structure is limitedly used for large-area displays. On the other hand, the top-emission structure, consisting of a completely reflective bottom electrode and a (semi-)transparent top electrode, emits light in the opposite direction of the substrate. Thus, it guarantees light emission from the full-subpixel area without any impediments, leading to being commonly applied for small-sized displays. No substrate in the optical path may considerably improve the light outcoupling efficiency [89,90,91]. Also, a microcavity effect between two metallic electrodes can be utilized to tune the peak wavelength and enhance the color purity [92,93,94,95]. However, intense light emission in the normal direction results in a distorted angular profile of the Lambertian distribution, which may cause a poor viewing angle in display devices. Recently, a highly efficient top-emitting QLED with an angular-independent emission profile was demonstrated by introducing a nanosphere scattering layer [96]. Also, a high external quantum efficiency (EQE) of 32.5% was achieved by enhancing the light extraction efficiency owing to the reduced absorption and waveguide losses and minimizing the surface plasmon polariton mode at the metal–dielectric interface. More studies on top-emitting QLEDs are needed, considering the significance of this topic to the industry.

A tandem structure, composed of two or more EL units connected in series, is also applicable to QLEDs. Since a QLED is driven by current, an electrical series connection of them enables the operation of all the EL stacks at the same current level. Thus, the EQE and current efficiency tend to increase roughly in proportion to the number of EL units, but the driving voltage increases so that the power efficiency remains the same or slightly lower due to increased internal resistance [97,98,99,100]. Between each EL unit, a charge generation layer (CGL) or an interconnecting layer should be formed, in which electrons and holes are generated and supplied to adjacent two units, respectively. For that, the CGL generally comprises a planar heterojunction (e.g., p–n junction or p–metal junction) for efficient charge separation with properties of low resistance and high transparency. Particularly in QLEDs, the CGL deposition process needs to be compatible with the fabrication process of each EL unit, which makes it hard to stack multiple QLED units.

### 3.2. High-Performance QLEDs Based on Device Engineering

**Figure 4:**
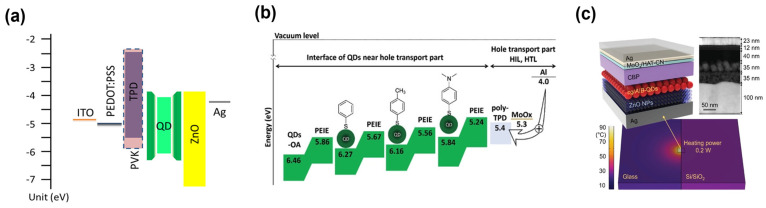
Device engineering methods: (**a**) Composite of PVK and TPD as the HTL for the QLED (Reproduced from [105] with permission from American Chemical Society). (**b**) Energy band diagram of the green QLED with interface-modified QDs (Reproduced from [122] with permission from Wiley). (**c**) Schematic illustration of an inverted top-emission QLED fabricated on substrates with a different heat dissipation property (Reproduced from [123] with permission from Wiley).

Within a certain QLED structure, we can further modify the properties of each constituting layer to improve device performance. In particular, poor charge balance is one critical issue in this research field, which can be resolved mainly by device engineering. For this, various approaches and methods have been suggested, such as doping or blending a charge transporting layer (CTL) or insertion of an additional layer, and QD surface treatment. First, changing the properties of the CTL by doping or mixing an additional material is an intuitive method. As mentioned above, there have been difficulties in hole injection into QDs, leading to a large number of researches on HTL modification. Doping the HTL with an electron acceptor, e.g., 2,3,5,6-tetrafluoro-7,7,8,8-tetracyanoquinodimethane (F4-TCNQ) and MoO_x_, improves hole conduction ability and further enhances the thermal stability of the HTL [101,102]. A composite HTL serves complementary properties of two materials in terms of energy level, mobility, and morphology, as shown in Figure 4a. In detail, small molecule–polymer composites typically exhibit good mobility from a small molecule in a robust polymer matrix. Representative small molecules for this purpose are 4,4′-bis(N-(3-methylphenyl)-N-phenylamino)biphenyl (TPD), 4,4′,4-tris(carbazol-9-yl)triphenylamine (TCTA) and 4,4′,N,N′-diphenylcarbazole (CBP), and widely-used matrix polymers are poly[N,N′-bis(4-butylphenyl)-N,N′-bis(phenyl)-benzidine] (poly-TPD), poly(9,9-dioctylfluorene-alt-N-(4-sec-butylphenyl)-diphenylamine) (TFB), and poly-N-vinylcarbazole (PVK) [103,104,105,106,107,108,109,110,111].

The properties of the ETL can also be modified by doping and additives. For instance, ZnO has good electrical properties for QLEDs but has defect states from intrinsic oxygen vacancy, resulting in device instability and severe exciton quenching. Metal-doped/alloyed ZnO, such as ZnMgO and Li/Al/Ga-doped ZnO, has been reported to show improved device performance of QLEDs [112,113,114,115]. Inserting an additional layer has also been demonstrated for high-performance QLEDs. Especially, the insertion of a poly(methylmethacrylate) (PMMA) layer near the QD layer effectively blocks leakage current through the QD layer to obtain high EQE [116,117]. Similar effects were reported by incorporating a thin insulating layer (e.g., Al_2_O_3_, polyethylenimine ethoxylated) and a thin semiconducting organic layer having low mobility and wide bandgap [118,119,120,121].

Modification of the QD surface with proper ligands is also an important strategy for achieving high performance because insulating surfactants surrounding the QDs can hinder charge transport. A few methods have been reported to improve the optoelectronic properties by QD surface modification, such as ligand exchange with shorter ligands, electron donating/accepting ligands, and semiconducting ligands. Ligands with shorter alkyl chains or smaller molecules instead of oleic acids may provide dense, uniform, and stable packing of QDs, resulting in enhanced carrier injection/transport in QLEDs [122,124,125,126,127]. Adopting the ligand with a strong dipole moment originating from the electron donor/acceptor group can shift the energy level of QDs, which is helpful for balancing charge injection into QDs. For example, 2-ethylhexane-1-thiol ligands upshifted the energy level of Zn_1-x_Cd_x_Se/ZnSe/ZnS QDs and reduced the hole injection barrier, resulting in a current efficiency of 72 cd A^−1^, the maximum luminance of 334,000 cd m^−2^, and a half-lifetime of 140 h at an initial luminance (*L*_0_) of 17,200 cd m^−2^ which corresponds to that of 1,800,000 h at *L*_0_ of 100 cd m^−2^ by estimation [8].

Also, precise tuning of the energy level of QDs was demonstrated by ligand exchange with the series of thiophenol derivatives having a negative dipole moment, as shown in Figure 4b [122]. As a result, a green QLED exhibited improved current efficiency and maximum luminance of 98.2 cd A^−1^ and 106,400 cd m^−2^, respectively. Furthermore, QD surface treatment with halide anions can be a promising approach to enhance the QLED performance [14,113,125]. Ligand exchange and treatment with halides have effects in improving charge transport properties and reducing defects of QDs. Instead of insulating ligands, semiconducting ligands, which have a good charge transport property, have also been of interest for charge balance in QLEDs [126,127]. For the semiconducting property, the ligands typically contain bulkier functional groups in the form of oligomers or polymers. Thus, the QD film shows increased dot-to-dot distance but improved charge transport ability. Furthermore, it leads to the widening of the exciton recombination zone in the thicker emitting layer, which is ideal for reducing the efficiency roll-off.

Upon engineering the internal device structure, the introduction of a new substrate having a high thermal conductivity has recently been of interest in reducing thermal degradation. Joule heating is unavoidable in current-driven QLEDs, which deteriorates the efficiency and stability of the devices. Low thermal conductivity of the widely-used glass substrate (~1.0 W m^−1^ K^−1^) hinders heat dissipation and thereby increases the temperature of subpixels, which is known to accelerate irreversible thermal degradation of organic HTLs and QD ligands. To resolve this issue, Sun et al. employed a sapphire substrate with high thermal conductivity of 46 W m^–1^ K^–1^ and a thermally stable ligand, 1-dodecanethiol [12]. As a result, a high current efficiency of 75.3 cd A^−1^ and maximum luminance of 1,680,000 cd m^−2^ have been achieved in a green QLED. Recently, Lee et al. also demonstrated highly bright and stable QLED using a Si/SiO_2_ substrate with high thermal conductivity of ~150 W m^–1^ K^–1^ [123], as shown in Figure 4c. Based on additional device optimization, the QLED on the Si-based substrate exhibited a current efficiency and maximum luminance of 75.6 cd A^−1^ and 3,300,000 cd m^−2^, respectively.

### 3.3. QD Patterning Technologies

In order to realize AM-QLEDs, the QD layer should be formed in each individual subpixel. For this, QD patterning technologies have been investigated to comply with the requirements depending on the types of display application in terms of size, resolution, and the number of colors. In this section, we briefly introduce a few methodologies for QD patterning, such as contact printing, inkjet printing, photolithography, and light-induced direct QD patterning, and their pros and cons.

**Figure 5:**
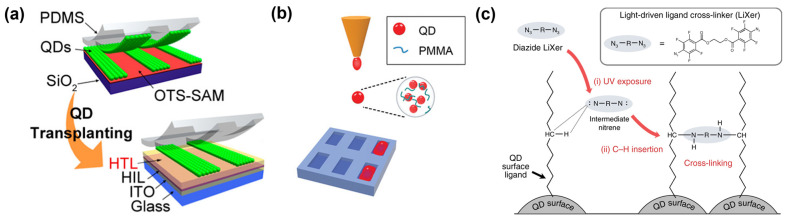
Schematic illustration of various QD patterning methods: (**a**) Contact transfer printing of QDs using a PDMS stamp (Reproduced from [129] with permission from American Chemical Society). (**b**) Inkjet printing of QD solution into subpixels (Reproduced from [130] with permission from Wiley). (**c**) UV-induced ligand crosslinking process for direct patterning of the QD film (Reproduced from [131] with permission from Springer Nature).

First, contact printing or contact transfer printing is one of the simplest patterning methods using an elastic stamp. A QD film deposited on a pre-patterned stamp can be transferred onto a target surface by using the difference of adhesion. In 2008, Kim et al. first reported a contact-printed QLED with a resolution of 1000 pixels-per-inch (PPI) using a Parylene-C-coated poly(dimethylsiloxane) (PDMS) stamp to control the surface energy and to prevent it from swirling by the QD solvent [128]. In 2011, Kim et al. introduced a self-assembled monolayer on the master substrate for easier detachment of the QD film and demonstrated a full-color AM-QLED [18]. Contact printing requires a high pressure to assure the transfer of QDs, and thus it is likely to damage the underlying layers and the transferred QD layer as well. In 2015, Cho et al. developed a soft contact QD transplanting technique, enabling the transfer of a QD layer onto a soft organic layer without pressure [129], as shown in the Figure 5a. Nevertheless, contact printing with a stamp is not desired in the industry because of several issues in printing accuracy, stamp reliability, limited printable area, and non-uniformity.

As a promising patterning technology, particularly for full-color AM-QLED displays, inkjet printing is attracting great attention from both academia and industry, owing to its high accuracy and low material usage based on drop-on-demand, and high throughput for large-area printing. On the other hand, poor film morphology is a weakness of inkjet printing. Inkjet-printed film morphology is related to several factors, such as the boiling point and vapor pressure of the solvent, the viscosity of ink, the surface energy of the substrate, and drying conditions. In most early-stage inkjet-printed QLEDs [132,133,134], the efficiency and brightness were relatively lower than those of the spin-coated QLED devices, mainly attributed to the poor QD morphology. Several strategies have been introduced to overcome this issue, such as using co-solvents to tune the boiling point and vapor pressure, mixing additives to control the viscosity, and substrate surface treatment to enhance wettability [130,135,136,137,138,139,140]. The Marangoni and capillary forces in a droplet can be optimized using these techniques to obtain a high-quality film. For example, Roh et al. reported QD–PMMA composite inks in a co-solvent to control the aforementioned key parameters and inkjet printing of fine QD films [130], as shown in Figure 5b. A ternary ink system consisting of octane, 1-cyclohexyl-ethanol, and n-butyl acetate was adopted to improve the morphology of QD film [15]. Recent intensive research on inkjet-printed QLEDs combined with device engineering has brought the inkjet-printed QLED performance to a level comparable to spin-coated ones [108,130,140,141], which should ease the commercialization of QLED displays.

Traditional photolithography can also be applied to pattern each QD subpixel. This matured technology provides a few advantages, such as high-resolution patterning and compatibility with the backplane fabrication procedure. However, there are several problems, for instance, physical damage to underlying QD and organic layers during photoresist (PR) deposition, development and removal processes, degradation of QDs by UV exposure, and defect formation from PR residues. Although full-color, high-resolution QLEDs could be demonstrated based on photolithography, the procedure to minimize the damages is exceedingly complicated [142,143]. Recently, a direct QD patterning method has been reported, which is a technique for patterning QDs based on photolithography but without the PR processes [131,144,145,146,147,148]. It can be achieved by adopting light-responsive ligands onto the surface of QDs, which chemically binds QDs upon illumination to firmly hold the pattern against the following wash-out step.

Specifically, in 2020, Cho et al. introduced direct optical lithography based QLEDs by employing photosensitive surface ligands, showing selective, full-color QD patterning with a small feature size of ~1.5 µm [146]. Yang et al. also reported a QD direct patterning method using a light-driven ligand crosslinker [131]. UV exposure to the modified QD film induces a chemical reaction between azides and the alkyl chain of QD ligands, resulting in a crosslinked QD layer, as shown in Figure 5c. Full-color, high-resolution QD patterns of >1400 PPI were demonstrated, and red crosslinked QLEDs exhibited an EQE of 14.6%. In 2022, Hahm et al. proposed direct patterning with dual ligands composed of dispersing ligands and photo-cross-linkable ligands to maintain the PL QY during photo-crosslinking [148]. As a result, they successfully demonstrated full-color QLED arrays employing the patterned QD films in a high resolution of >15000 PPI without a performance loss. Nevertheless, both traditional and direct photolithography requires a conventional UV lithography system for alignment and UV exposure, and thereby the processible substrates that can be processed are limited in size to the current wafer scale (300 mm). Thus, it is expected to be narrowly utilized for small-sized, high-resolution display devices, such as augmented/virtual reality (AR/VR) display devices.

Above and beyond these technologies, more QD patterning technologies, such as 3D printing [149,150], electrohydrodynamic printing [151], phase separation [152], electrophoretic deposition [153], and spatial light-assisted surface tailoring [154], have been introduced. However, these technologies are not suitable for implementing high-resolution displays and multilayer stacking or require complicated processes. Instead, we think that these technologies can be used in other ways, for instance, the fabrication of a QD color conversion layer for low-resolution displays or small lighting devices.

## 4. Monolithically Integrated TFT–QLED Devices

In Section 2 and Section 3, we reviewed the development of TFT and QLED devices, respectively, in terms of circuitry design and electrical requirements for reliable AM driving. However, monolithic integration of TFT–QLED requires the use of compatible processes to achieve high performance for both devices in a concise and effective TFT–QLED design with a tolerable fabrication procedure to each other. Thus, we think that it is important to investigate the fabrication process, optical and electrical properties, and any possible combinational issues in monolithically integrated TFT–QLED devices.

**Figure 6:**
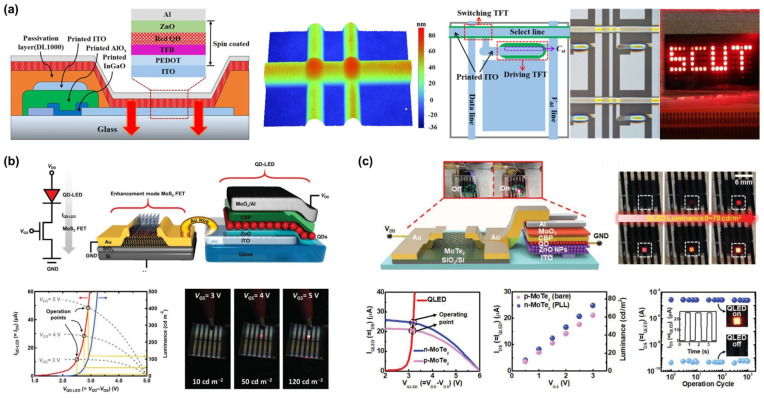
Monolithically integrated TFT–QLED devices: (**a**) Schematic cross-sectional diagram of inkjet-printed TFT–QLED pixel, the morphology of the inkjet-printed oxide film, the pixel structure, and a demonstration of the 2T1C-structured AM-QLED (Reproduced from [24] with permission from American Chemical Society). (**b**) Schemes of TFT–QLED using the multilayered MoS_2_ TFT with ODTS-SAM on the back channel, current–voltage characteristics along with the driving current level, and luminance change (Reproduced from [23] with permission from Wiley). (**c**) Schematic structure of MoTe_2_ TFT-driven QLED with luminance changes, current–voltage characteristics of type-converted TFTs via PLL treatment, current–voltage–luminance characteristics of the TFT–QLED device, and their stability (Reproduced from [25] with permission from Wiley).

As reviewed above, an oxide TFT is one of the promising backplane devices, as already used in the industry. They also have large-area processability and high electrical performance sufficient to drive QLEDs. Recently, Chen et al. showed that the luminance of an inverted QLED could be competently controlled by an IGZO TFT [26]. In particular, they developed Al reaction-induced conductive a-IGZO as a common electrode, exhibiting a low sheet resistance (120 Ω sq^–1^) and a high transmittance (92.1%), demonstrating reduced photolithographic mask steps as well as higher power efficiency at the same time. In 2019, Li et al. showed QLED arrays integrated on inkjet-printed InGaO TFTs [24], which is suitable for large-area AM-QLED fabrication. To improve the quality of inkjet-printed oxides, they adopted a solvent printing method and demonstrated red-emitting QLED arrays driven by a 2T1C circuit, as shown in Figure 6a.

Owing to the remarkable development of TFTs using TMDCs as an active material, integration of the TMDC-based TFTs with QLEDs has been presented gradually. In 2019, Roh et al. reported an enhancement-mode n-type MoS_2_ TFT by octadecyltrichlorosilane (ODTS) SAM treatment to drive inverted QLEDs [23], as shown in Figure 6b. A deep-seated problem in depletion-mode TFTs is the large gate overdrive voltage ensuing rapid degradation of the channel. But the ODTS SAM enabled MoS_2_ TFTs to operate in an enhancement mode, leading to depopulate electrons on the back channel. As a result, they showed a wide-range operation of the QLEDs by controlling *V*_T_. In 2021, Baek et al. also demonstrated the stable operation of QLEDs driven by an integrated type convertible MoTe_2_ TFT [25], as shown in Figure 6c. In particular, they showed that the TFT–QLED integrated device exhibits a negligible response to visible light and a fast response time utilizing the properties of the MoTe_2_ channel. Also, a type-convertibility of the TFTs by simple poly-L-lysine (PLL) treatment was demonstrated, which allows using both normal and inverted structures of QLEDs.

As reviewed in this section, the research on TFT–QLED integrated devices is in its early stages but is gradually on the increase. The development of AM-QLED should be accompanied by not only respective performance improvement of the TFT backplane and QLED frontplane but also the requirements for monolithically integration in terms of circuitry design, the proper combination of TFT–QLED, and facile fabrication method. Such interest and intensive research would contribute to overcoming the known issues and limitations in regard to the fabrication process and reliability, enabling the realization of AM-QLEDs with advanced features.

## 5. Conclusions

In this review, we presented the fundamentals, state-of-the-art performance, and prospects of non-Si-based TFTs, QLEDs, and their combined forms, to achieve AM-QLED displays. As channel materials of the backplane TFT, metal oxides, TMDCs, and semiconducting SWNTs were considered to provide the requirements (e.g., high current driving, small *V*_T_ shift under BTS) for future display devices. Their new features against Si, such as solution-processability, light-insensitivity, flexibility, and transparency, may lead to a strong chance of success in a display application, although there still exist several issues (e.g., performance, stability, and solution-based fine patterning) which need to be resolved. Examination of QLEDs from the viewpoint of device architecture, device engineering, and QD patterning is also meaningful, giving a new prospective on the future research direction. Although the QLEDs have been improved continuously, it is required to further develop more stable devices based on Cd-free QDs. In addition, the performance of pixelated QLEDs needs to be enhanced more. Finally, we reviewed the monolithic integration of TFT–QLED devices with the findings and issues toward AM-QLEDs. By solving the present issues, TFT-driven AM-QLEDs can be widely used as the main display panel with the advantages of a wide color gamut, high brightness, and low power consumption, which would expand the practical use from mobile devices and TVs to outdoor displays and AR/VR devices. We believe that this comprehensive review would be a valuable and inspiring guide for the readers in their future research with a wider perspective on AM-QLED displays.

## Data Availability

Not applicable.
